# Apical delta filling with calcium silicate and epoxy resin sealers using different obturation techniques by microcomputed tomography

**DOI:** 10.1590/1678-7765-2025-0774

**Published:** 2026-03-23

**Authors:** Washington Soares dos Santos, Jefferson Augusto, Angelo José Sócrates Torres-Carrillo, Igor Bassi Ferreira Petean, Antonio Miranda da Cruz-Filho, Manoel Damião de Sousa-Neto, Fabiane Carneiro Lopes-Olhê, Jardel Francisco Mazzi-Chaves

**Affiliations:** 1 Universidade de São Paulo Faculdade de Odontologia de Ribeirão Preto Departamento de Odontologia Restauradora Ribeirão Preto SP Brasil Universidade de São Paulo, Faculdade de Odontologia de Ribeirão Preto, Departamento de Odontologia Restauradora, Ribeirão Preto, SP, Brasil.

**Keywords:** 3D printing, Calcium silicate cement-based sealers, Micro–computed tomography, Obturation, Apical delta

## Abstract

**Objectives:**

This study aimed to evaluate the filling quality of simulated apical deltas in 3D-printed tooth replicas using microcomputed tomography and comparisons of two obturation techniques and calcium silicate cement-based sealer groups.

**Methodology:**

A single-rooted, straight maxillary premolar was instrumented and scanned by microcomputed tomography to generate a 3D model. Apical delta configurations were digitally created and integrated into the canal anatomy. In total, 40 translucent resin replicas were 3D printed and randomly assigned for obturation with one of four sealer groups (AH Plus Resin, AH Plus Bioceramic, Bio-C Sealer, and NeoSealer Flo). Following obturation, all specimens were rescanned, and a volumetric analysis was performed to determine the percentage of the filled volume in the apical delta region. Statistical analyses included one-way ANOVA and the Kruskal-Wallis test (α=0.05).

**Results:**

The continuous wave technique resulted in significantly greater apical delta filling than the single-cone one regardless of sealer. For the single-cone technique, NeoSealer Flo showed the highest filling percentage (42.7±2.0), followed by Bio-C Sealer (28.7±1.1), AH Plus Resin (24.9±1.9), and AH Plus Bioceramic (17.9±1.0). For the continuous wave technique, Bio-C Sealer showed the most filling (66.2±2.0), followed by NeoSealer Flo (54.2±1.4), AH Plus Resin (45.9±1.5) and AH Plus Bioceramic (36.8±1.0).

**Conclusions:**

The continuous wave technique achieved the most apical filling, with Bio-C sealer showing the highest performance. Bio-C and NeoSealer Flo achieved significantly higher filling percentages than AH Plus Resin and AH Plus Bioceramic under the single-cone technique.

## Graphical abstract

**Figure:**
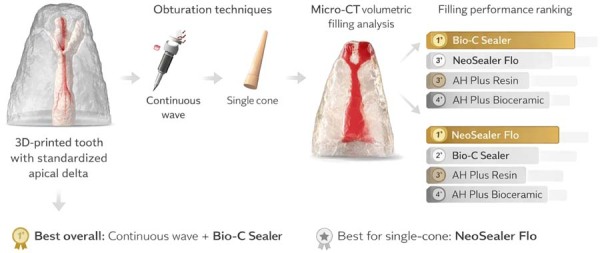
*Co-first authors

## Introduction

The intricate three-dimensional anatomy of the root canal system often includes accessory and lateral canals and apical deltas, which offer significant clinical challenges for effective disinfection and obturation.^1,2^ Apical deltas consist of multiple small ramifications near or at the root apex that diverge from the main canal.^3^ Microcomputed tomography (micro-CT) studies have confirmed the presence and variability of these structures, including in molars, mandibular incisors, and canines, with a prevalence of up to 12% depending on the anatomical classification criteria. These observations reinforce the need for improved obturation approaches in complex apical morphologies.^4^ Recent experimental data has shown that calcium silicate cement-based sealers can significantly fill more of the volume of lateral canal-like structures than epoxy resin-based ones, adding filling effectiveness beyond the insertion technique alone. This sealer composition is very important for the effective obturation of complex canal ramifications.^5^

Apical ramifications are often inaccessible to endodontic instruments and irrigants, potentially harboring necrotic tissue and residual microorganisms that may contribute to persistent periapical inflammation and endodontic failure.^6^ While the clinical significance of unfilled lateral canals has been questioned,^2^ histopathological evidence has shown their potential pathogenicity in incomplete healing.^7,8^ Ricucci and Siqueira Jr.^9^ (2010) have highlighted that filling materials often failed to reach these narrow extensions, raising concerns about long-term outcomes in anatomically challenging cases.

Sealer penetration into intricate areas such as accessory canals, isthmuses, apical deltas, and dentinal tubules is essential for achieving effective seals and enabling their antibacterial properties to contribute to reinfection control.^5^ These structures are often undetectable in preoperative imaging and may compromise cleaning and sealing procedures. Clinically, the presence of an apical delta should be suspected when the main canal becomes indistinct in the apical third and if working length determination is difficult. Post-obturation radiographs may occasionally reveal these regions, especially when radiopaque sealers or thermoplasticized gutta-percha are used. This complexity highlights the need to evaluate obturation techniques and sealer performance in anatomically challenging regions.^9^

Root canal sealers fill the interface between the canal wall and gutta-percha, reduce microleakage, and entomb residual bacteria,^10^being particularly relevant in irregular canal systems and apical deltas.^5,7,8^ Recent micro-CT analyses^5^ have suggested that sealer performance in lateral canals depends on sealer flow, viscosity, and particle size. Bio-C Sealer have shown enhanced performance in such conditions due to their ability to set in the presence of moisture.^5^ Various obturation strategies have been used to maximize sealer penetration, including cold lateral condensation, warm vertical compaction, and ultrasonic activation.^11^

The ideal endodontic sealer should combine adequate flow, dimensional stability, radiopacity, appropriate film thickness, low solubility, proper setting ability, biocompatibility, and antimicrobial efficacy.^12^ However, excessive flow may promote apical extrusion and periapical irritation.^13^ In Rosa-e-Silva, et al.^5^ (2025), no significant differences were observed in lateral canal filling between sealer insertion technique with a gutta-percha cone or an applicator tip; Bio-C Sealer consistently achieved higher filling percentages in all thirds of the root canal.

Calcium silicate cement-based sealers have garnered attention for their low cytotoxicity, bioactivity, alkaline pH, and calcium ion release.^5,14^ However, increased flowability has been associated with a potential risk of extrusion in certain clinical situations.^15^ Epoxy resin-based sealers, such as AH Plus, are widely recognized for their dimensional stability, adequate flow suitable for clinical use, good adhesion to dentin, and radiopacity. However, they lack bioactivity and calcium release and may show transient cytotoxic effects before complete setting.^16^

Numerous *in vitro* models have been employed to assess obturation quality in lateral canals. These models include natural teeth with artificial lateral canals assessed by radiography,^17^ teeth cleared with demineralization techniques,^18^ and standardized resin blocks.^19-21^

Although most studies have concentrated on lateral canals, no investigation has evaluated obturations in apical deltas. The use of 3D-printed replicas with standardized apical delta morphologies offers an innovative and reproducible approach to overcome the limitations of anatomical variability in experimental models.^22,23^ Combined with high-resolution micro-CT, non-destructive and reliable imaging can be used for three-dimensional evaluations of endodontic obturation quality.^24,25^

Thus, this study aimed to assess filling ability in simulated apical deltas using two obturation techniques—continuous wave and single-cone—and four root canal sealers. The null hypothesis was that no significant differences would be found between the techniques or materials with respect to delta filling.

## Methodology

### Ethical approval and sample calculation

The local Research Ethics Committee (CAAE: 79392224.1.0000.5419) approved this study. A recently extracted maxillary premolar (removed for periodontal reasons) was obtained from the Tooth Biobank of the School of Dentistry of Ribeirão Preto, University of São Paulo.

Sample size calculation was performed using G*Power 3.1.9 (Heinrich Heine University, Düsseldorf, Germany) based on parameters reported by Rosa-e-Silva, et al.^5^ (2025). Considering four groups, an effect size of 0.65, α=0.05, and an F-test framework, the calculation indicated a minimum requirement of 40 specimens (n=10 per group).

### Preparation of the natural tooth and resin replica

The tooth was initially cleaned in an ultrasonic bath for five minutes. The external root surface was then cleaned using ultrasonic scalers (Profi II Ceramic, Dabi Atlante Ltda, Ribeirão Preto, SP, Brazil) and stored in a 0.1% thymol solution.

Macroscopic examination and radiographic evaluation of the tooth were performed using a digital phosphor plate system (Eagle PS, Dabi Atlante, Ribeirão Preto, São Paulo) in the orthoradial and mesioradial directions (Spectro 70X Electronic, Dabi Atlante, Ribeirão Preto, SP, Brazil). The tooth was accepted as a model because it was straight and fully formed root that had a single canal and no calcifications, resorption, restorations, or cracks.

Access to the pulp chamber was created using a #1 round bur (KG Sorensen, Barueri, SP, Brazil) under high-speed rotation with continuous water cooling. The root canal was irrigated with 2 mL of 1% NaOCl using a disposable syringe (Ultradent, South Jordan, UT, USA) and a 0.30 mm NaviTip needle (Ultradent). Canal scouting was performed using a #15 K-file (Dentsply Maillefer, Petrópolis, RJ, Brazil) until the tip was seen at the apical foramen using a surgical microscope (Opto Novus, São Carlos, SP, Brazil). The working length was established by subtracting 2.0 mm from the length to the apical foramen.

Biomechanical preparation was performed using a Reciproc R50.05 instrument (VDW GmbH, Munich, Germany) in reciprocating motion. Final irrigation included 2 mL of 17% EDTA (Biodinâmica, Ibiporã, PR, Brazil) for 5 minutes, followed by 5 mL of 1% NaOCl. The canal was dried using a Capillary Tip suction cannula (Ultradent Products Inc., USA) and dried with #50 absorbent paper points (Dentsply Maillefer, Petrópolis, RJ, Brazil).

The instrumented tooth was scanned using a micro-CT system (SkyScan 1174 v2, Bruker-microCT, Kontich, Belgium) with the following parameters: 50 kVp, 800 µA, 26.97 μm isotropic resolution, 360° rotation with 0.7° rotation steps, one frame per projection, and a 0.5 mm aluminum filter. Images were reconstructed on Nrecon, v1.6.6.0 (Bruker-microCT, Kontich, Belgium), yielding axial cross-sections of the internal root anatomy. A 3D stereolithographic (STL) model was generated via binarization on CTAn v1.18.8.0+ (Bruker-microCT, Kontich, Belgium).

A 3D model was generated using the “double-time cubes” algorithm and exported into an STL format. The model was visualized on CTVol, v2.3.2.1 (Bruker-microCT), supporting the design of the apical delta morphology.

**Figure 1:**
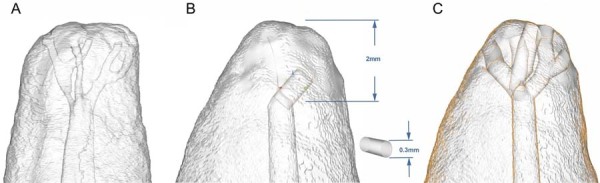
Apical delta modeling. (A) STL model designed based on Mazzi-Chaves et al. (2020). (B) Delta branches created using 300-μm-diameter cylinders. (C) Final apical delta morphology showing three branches ending in five apical foramina.

The STL file was imported into Blender^®^ 3D modeling software (Blender Foundation, Amsterdam, Netherlands). Using the model (Figure 1A) described by Mazzi-Chaves, et al.^4^ (2020), an apical delta was digitally designed with three branches ending in five foramina. Cylinders (300 μm diameter) were used for the delta channels and positioned within 2 mm from the apex using the “Measure” tool (Figure 1B). The “Add” and “Mesh” commands were also used. These cylinders were then duplicated and irregularly shaped to simulate apical ramifications, each containing five foramina (Figure 1C).

**Figure 2:**
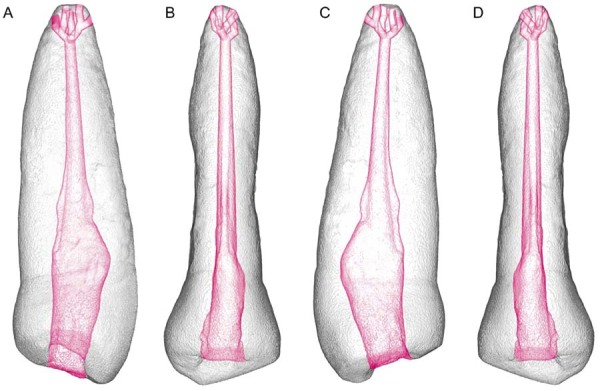
Visualization of the STL model of the 3D artificial teeth, with projections of the apical delta design on different surfaces. From left to right, the following views are shown: (A) mesial, (B) buccal, (C) distal, and (D) palatal. The root canal and the apical delta were shaded in red to enhance visualization.

The apical delta design was visually assessed in multiple three-dimensional planes to ensure that its extension reached the external root surface (Figure 2). The final STL model was then exported for 3D printing.

**Figure 3:**
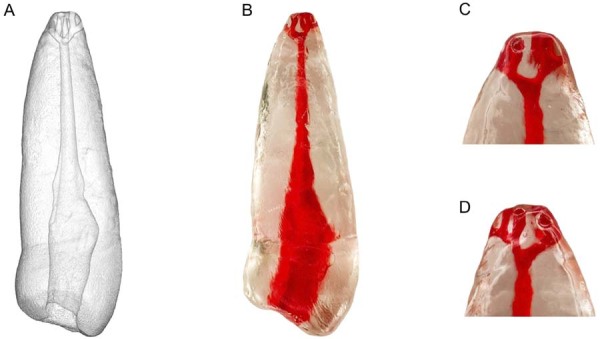
3D-printed replica. (A) STL file showing the apical delta morphology. (B) Replica printed in translucent resin, with the root canal and apical delta highlighted in red for visual enhancement. (C) Magnified view of the apical third highlighting the delta branches.

In total, 40 3D replicas were printed from the STL file using a FlashForge Hunter printer (FlashForge Corporation) with digital light processing technology and Prizma 3D Bio Guide resin (Makertech Labs, Tatuí, SP, Brazil) (Figure 3). Printing parameters included a 50-μm layer thickness, a 62.5-μm XY resolution, and a ±0.05-mm dimensional accuracy. The curing time was set at 3.5–4.0 s per layer, as were 20 s for adhesion layers, eight transition layers, and 90% light intensity on FlashDLPrint. After printing, replicas were washed in isopropyl alcohol for 5 min and UV-cured for 30 min to reduce resin shrinkage and artefacts. All replicas were generated from the same STL file to ensure consistent volume and geometry across the 40 samples.

To simulate periodontal tissues, an additional reference replica (printed from the same STL file but excluded from the experimental groups) was coated with type 7 modeling wax (Lysanda, São Paulo, SP, Brazil) at the apical region and positioned in a typodont socket (Orais Manequins Odontológicos, Goiânia, GO, Brazil). This replica only served to create an artificial socket. It was secured using type III dental stone (Vigodent-Coltene, Rio de Janeiro, RJ, Brazil) mixed with sawdust to radiographically simulate trabecular bone. After the stone had set, the reference replica and the apical wax were removed, creating a standardized socket. Each of the 40 printed 3D replicas was then individually placed into the socket for obturation, reproducing clinical handling conditions and simulating procedural difficulty, as described above.

### Root canal obturation

In total, 40 3D-printed replicas were divided into four groups (n=10) according to the used sealer:

Group I – AH Plus Resin (Dentsply Sirona, São Paulo, SP, Brazil)

Group II – AH Plus Bioceramic (Dentsply Sirona, São Paulo, SP, Brazil)

Group III – Bio-C Sealer (Angelus, Londrina, PR, Brazil)

Group IV – NeoSealer Flo (Avalon Biomed, Houston, TX, USA)

Each group was further subdivided (n=5) according to the obturation technique: the single-cone technique, using a size R50 gutta-percha cone (VDW GmbH, Munich, Germany), and the continuous wave (thermoplastic) technique, performed with the EQ-V Master system (MetaBiomed, Cheongju-si, South Korea). For the single-cone technique, an R50 gutta-percha cone was selected to match the final instrument size. The sealer was applied to the canal using a #40 K-file (Dentsply Maillefer, Petrópolis, RJ, Brazil), and the master cone was coated with sealer and inserted to the full working length without additional compaction.

For the continuous wave technique, the EQ-V Pack unit was preheated to 180°C. A size 50/.04 plugger was inserted to a position 3 mm short of the working length while activated. After softening the gutta-percha, the plugger was maintained in place for 10 seconds, then reactivated for 1 second and withdrawn, leaving the apical 3 mm from the gutta-percha. Apical cold compaction was performed using a #60 nickel-titanium hand plugger (Easy Equipamentos Odontológicos, Belo Horizonte, MG, Brazil). The remaining canal space was filled with thermoplasticized gutta-percha delivered by the EQ-V Fill device set to 200°C using a 25G needle.

Digital radiographs were recorded in orthoradial and mesioradial projections to assess cone fit and adaptation. All obturations were performed by a single experienced operator to ensure consistency. The specimens were randomly assigned to the experimental groups using a randomization procedure with an online random number generator (randomizer.org).

### Post-obturation micro-CT analysis

The 40 obturated replicas and one unfilled control were scanned using the SkyScan 1174 system (Bruker-microCT, Kontich, Belgium). A single pre-obturation scan of one replica was designated as the reference image, as all 3D-printed replicas shared identical anatomy. Each post-obturation dataset was dynamically registered to this reference using the “3D Registration” tool on Data Viewer v1.5.1.2 (Bruker-microCT, Kontich, Belgium). A single pre-obturation image from a non-obturated reference replica was designated as the reference, and all post-obturation images were dynamically registered to this reference as all replicas shared identical geometry. A new dataset representing the difference between the two scans (“Image Difference”) was generated and saved.

### Volumetric analysis of the apical delta by micro-CT

Following image alignment, the post-obturation scans were analyzed on CTAn v1.18.8.0+ (Bruker-microCT, Kontich, Belgium). Regions of interest were the apical third and the apical delta region. The most apical slice showing obturation was selected as the “Top” and the subsequent 3 mm in the coronal direction, as the “Bottom.” For the apical delta regions of interest, the most apical and coronal slices encompassing the delta morphology were selected.

Grayscale segmentation was performed using interactive thresholding, producing binary images with white pixels representing the region of interest and black pixels representing the background. A customized task list of plug-ins was used for image processing, and volume data (mm^3^) were recorded for each region of interest. Volume values were exported onto CTAn.

Filling volume was calculated by subtracting the pre-obturation volume from the post-obturation volume in each replica. The percentage of filled volume was then calculated based on the total canal volume and recorded. 3D renderings were created on CTVol v2.3.2.1 (Bruker-microCT) for enhanced visualization, with distinct colors assigned to the sealers and teeth.

### Qualitative and quantitative assessment

Overall, two calibrated examiners who were blind to the obturation technique and used materials independently evaluated the filling quality of the apical deltas using a five-point ordinal scale: (1) excellent, 100% filled; (2) good, 75–100% filled; (3) fair, 50–75% filled; (4) poor, 25–50% filled; and (5) absent, <25% filled. Their calibration was conducted by assessing 20% of randomly selected samples, yielding a Cohen’s kappa value of 0.791, indicating substantial inter-examiner agreement.

### Statistical analysis

To assess the influence of obturation technique (single-cone and continuous wave) and sealer type (AH Plus Resin, Bio-C Sealer, NeoSealer Flo, and AH Plus Bioceramic) on the percentage of filled volume, data are shown as means and standard deviations. Normality and homogeneity of variances were verified using the Shapiro-Wilk + (P>0.05) and Levene’s test (P>0.05), respectively. Data that met parametric assumptions were analyzed using two-way ANOVA, which included “sealer type” and “obturation technique” in their interactions. The Tukey’s *post hoc* test was applied for pairwise comparisons when significant differences were detected (P<0.05).

Non-parametric data (filling quality scores) were analyzed using the Kruskal-Wallis test with Dwass-Steel-Critchlow-Fligner *post hoc* comparisons (P<0.05). When appropriate, the Mann–Whitney U test was used to compare single-cone and continuous wave obturation techniques. Statistical analyses were performed on Jamovi, v1.6.23 (The Jamovi Project, Sydney, Australia) with the level of significance set at 5%.

## Results

The percentage of filled volume in the apical delta region varied significantly according to sealer type and obturation technique. The continuous wave technique showed superior filling than the single-cone one regardless of the sealer. Results are shown in Table 1. With the single-cone technique, NeoSealer Flo showed the highest mean filling percent (42.7±2.0), followed by Bio-C Sealer (28.7±1.1), AH Plus Resin (24.9±1.9), and AH Plus Bioceramic (17.9±1.1). When the continuous wave technique was used, Bio-C Sealer showed the most filling (66.2±2.0), followed by NeoSealer Flo (54.2±1.4), AH Plus Resin (45.9±1.5), and AH Plus Bioceramic (36.8±1.0), as in Table 1.

**Table 1:** Means and standard deviations of filled volume (%) according to sealer group and obturation technique.

	AH Plus	AH Plus Bioceramic	Bio-C Sealer	NeoSealer Flo
Single Cone	24.90±1.94^Cb^	17.90±1.09^Db^	28.70±1.12^Bb^	42.70±1.17^Ab^
Continuous Wave	45.90±1.52^Ca^	36.80±0.99^Da^	66.20±1.97^Aa^	54.20±1.44^Ba^

Different uppercase letters indicate statistically significant differences between obturation techniques in the same sealer (row-wise). Different lowercase letters indicate significant differences between sealers in the same technique (column-wise) (Tukey post hoc test, P<0.05).

Overall, the two-way ANOVA showed the statistically significant effects of sealer type, obturation technique, and their interaction (all P<0.001; Table 1).

Pairwise *post hoc* analysis of the interactions between sealer and obturation technique was conducted to find specific group differences (P<0.05). In all comparisons, the continuous wave technique resulted in significantly higher filling percentages than the single-cone technique regardless of the used sealer.

### Qualitative filling score analysis

Table 2 shows the percentage distribution of qualitative filling scores for each combination of sealer and obturation technique. Even the best performing combinations, Bio-C Sealer and NeoSealer Flo with the continuous wave technique, achieved only the “fair” category (score 3: 50–75% filled), highlighting that apical delta filling remained incomplete and often suboptimal under all conditions. AH Plus Resin and AH Plus Bioceramic, when used with the same technique, showed poor filling outcomes as 100% of specimens scored 4 (poor: 25–50% filled).

**Table 2:** Percentage distribution of filling scores for each sealer group in the apical delta region.

Sealer	Technique	Score	Percentage
AH Plus	Single cone	5	60%
		4	40%
	Continuous wave	4	100%
AH Plus Bioceramic	Single cone	5	100%
	Continuous wave	4	100%
Bio-C Sealer	Single cone	4	100%
	Continuous wave	3	100%
NeoSealer Flo	Single cone	4	100%
	Continuous wave	3	100%

Filling score criteria for the apical delta region: Excellent – 100% of the delta area filled; Good – 75% to <100% of the delta area filled; Fair – 50% to <75% of the delta area filled; Poor – 25% to <50% of the delta area filled; Absent – <25% of the delta area filled.

The Kruskal-Wallis test showed statistically significant differences in apical delta filling scores in the tested sealers (P<0.01). Pairwise comparisons indicated that Bio-C Sealer and NeoSealer Flo achieved significantly better filling scores than AH Plus Resin and AH Plus Bioceramic. The Kruskal-Wallis test also showed that the continuous wave technique resulted in higher filling scores than in the single-cone technique.

**Figure 4:**
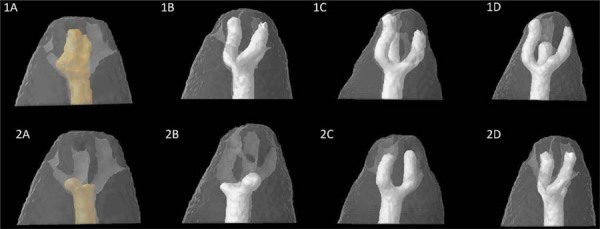
Representative 3D models of apical delta filling obtained using several obturation techniques and root canal sealer groups. 1A. Continuous wave technique with AH Plus Resin. 2A. Single-cone technique with AH Plus Resin. 1B. Continuous wave technique with AH Plus Bioceramic. 2B. Single-cone technique with AH Plus Bioceramic. 1C. Continuous wave technique with Bio-C Sealer. 2C. Single-cone technique with Bio-C Sealer. 1D. Continuous wave technique with NeoSealer Flo. 2D. Single-cone technique with NeoSealer Flo.

Figure 4 shows micro-CT images illustrating the apical delta obturation of 3D-printed replicas using two obturation techniques and various types of root canal sealers.

## Discussion

Calcium silicate cement-based sealers are widely used in endodontics due to their biological and physicochemical properties.^5^ Their higher flow when compared to AH Plus supports their application with the single-cone technique. However, heat during warm obturation can evaporate the organic liquid in some formulations, creating the impression of faster setting and altering stability.^26^ This effect varies across sealers as they fail to share the same organic medium.

Several studies have shown that thermoplastic obturation techniques can enhance the quality of obturations when used with calcium silicate cement-based sealers.^27-29^ The current findings are consistent with those observations. The application of heat via the continuous wave technique failed to compromise sealer performance; rather, it significantly improved apical delta filling. These results suggest that controlled heat promotes material flow and adaptation, highlighting its role in optimizing sealer behavior rather than impairing it. This challenges the assumption that thermoplastic techniques are inherently detrimental to calcium silicate sealer properties. Based on the results, the null hypotheses in this study have been rejected.

These results agree with a recent micro-CT study by Rosa-e-Silva, et al.^5^ (2025) who have shown that calcium silicate cement-based sealers, particularly Bio-C Sealer, achieved superior filling of simulated lateral canals than AH Plus, a resin-based sealer. In that study, tridimensional obturation was favored even under the single-cone technique, emphasizing the role of sealer flowability and its interaction with anatomical complexities such as lateral canals and deltas. Mazzi-Chaves, et al.^4^ (2020) reported an apical delta prevalence of 12% in human teeth using high-resolution micro-CT and highlighted the relevance of classification systems for detecting and interpreting these anatomical features. Their research supports advanced imaging and *in vitro* standardization (such as 3D-printed replicas) for evaluating obturation strategies in challenging apical anatomies. The findings in this study show that obturation techniques and sealer type directly influence the filling effectiveness in simulated apical deltas.

In this study, the EQ-Master system was set to 230°C as reported by Atmeh, et al.^30^ (2020). However, the actual temperature at the plugger tip was approximately 60°C. Epoxy resin–based and most calcium silicate cement-based sealers are considered thermally stable at temperatures below 100°C. Nevertheless, this may fail to apply to all formulations in the literature, such as BioRoot RCS. The protocol in this study respected this safety margin to preserve the integrity of its materials.

NeoSealer Flo achieved the highest filling percentage under the single-cone technique, followed by Bio-C Sealer, AH Plus Resin, and AH Plus Bioceramic. This superior performance may be related to its physical characteristics, which favor flow and adaptation. With the continuous wave technique, Bio-C Sealer showed the best results. However, this cannot be attributed solely to formulation as other factors such as thermal behavior may contribute.^28^ Flow characteristics should also be considered as studies using ISO 6876:2012 standards have shown that NeoSealer Flo and Bio-C Sealer meet the recommended flow requirements, whereas some bioceramic formulations, such as NeoMTA2, show lower flow values.^31^ Conversely, AH Plus Resin consistently showed the lowest filling values in both techniques, which could be associated with its limited flow characteristics. These findings reinforce the importance of flowability in optimizing sealer adaptation to complex apical anatomies.^32,33^

This study used 3D-printed replicas to ensure methodological standardization based on a single natural premolar scanned by micro-CT. Following protocols from previous investigations,^23,34,35^ the tooth was instrumented before scanning to achieve uniform biomechanical preparation and prevent resin debris from accumulating in the apical delta branches, which could occur if instrumentation is not performed beforehand.

According to Gao, et al.^36^ (2016), the mean diameter of apical delta branches is approximately 132µm, with a maximum reported diameter of 660 µm and a mean vertical distance of 1.9 mm from the apex. In this study, the simulated delta was designed with a 300-µm diameter and positioned 2 mm from the anatomical apex considering current 3D printing resolution (XY resolution of 62.5 µm and 50-µm layer thickness) and the voxel size of the micro-CT scan. A primary limitation of this study is that the 3D-printed replicas may fail to fully reproduce the complexity of natural apical deltas. Additionally, the resin material used for the replicas fails to replicate the microstructure of natural dentin, including the presence of dentinal tubules, which may influence sealer penetration and adaptation. Another limitation refers to its relatively small sample size (n = 5 per subgroup), which may have reduced its statistical power. Although the sample size was calculated a priori on G*Power, future studies with larger samples are recommended to confirm these findings.

The type of obturation technique had a strong interaction with sealer performance. The performance of these sealers was technique-dependent, a concept emphasized by Camilleri^37^ (2015). Notably, the continuous wave technique had a greater effect on filling outcomes than the choice of sealer.

The selection of the sealer remains relevant because Bio-C Sealer and NeoSealer Flo performed better than AH Plus bioceramic. The best fills were achieved when Bio-C Sealer was combined with the continuous wave technique, although significantly indifferent from NeoSealer Flo. These findings highlight the importance of considering the intrinsic properties of materials and their compatibility with the chosen obturation method. As this is an *in vitro* study, its findings should not be directly extrapolated to clinical practice. Further research under more clinically representative conditions is warranted.

Although certain combinations of sealers and techniques yielded better results, the overall filling of the apical delta region remained limited. Most specimens fell into unsatisfactory performance categories. While Bio-C Sealer and NeoSealer Flo performed better particularly when used with the continuous wave technique, no tested combination achieved complete or near-complete filling of the apical delta system.

Another limitation of this study lies in the interaction between calcium silicate cement-based sealers and the 3D-printed resin material, which fails to fully replicate the properties of natural dentin. In this *in vitro* model, canals were kept dry after irrigation without additional moisture simulation. Bio-C Sealer, combined with the continuous wave technique, best filled the apical deltas, followed by NeoSealer Flo under the same technique. These results partially align themselves with findings by Nawar, et al.^38^ (2024), who evaluated Bio-C Sealer and other calcium silicate–based sealers, reporting that controlled drying using paper points without desiccation improved interfacial adaptation. Conversely, Pelozo, et al.^39^ (2023) observed greater bond strength for Bio-C Sealer in slightly moist canals, with no significant differences in interface quality when compared to dry conditions. This standardized model ignored direct assessments of setting; therefore, the absence of moisture cannot be interpreted as having no effect on hydration. However, the observed filling performance suggests that these materials can still effectively adapt under controlled experimental conditions even without simulated moisture.

The findings of this study provide insight into the obturation of anatomically complex regions such as apical deltas and highlight the relevance of testing advanced obturation techniques, such as the continuous wave method, with calcium silicate cement-based sealers. Although delta filling remains a challenge, the observed improved performance with the continuous wave technique (especially when used with Bio-C Sealer and NeoSealer Flo) suggests that three-dimensional sealing of complex apical morphologies may be optimized by the synergy between sealer and technique. Thermoplastic obturation methods are suitable for cases under suspected complex apical anatomy. The development and validation of a standardized 3D-printed tooth model with reproducible apical deltas was a useful *in vitro* approach to compare materials and techniques. This study underscores the importance of evidence-based testing of obturation protocols to improve outcomes in challenging clinical scenarios.

## Conclusions

This *in vitro* study with identical tooth models showed that combining the continuous wave technique with Bio-C Sealer obtained the most effective approach for filling apical deltas. When the single-cone technique is preferred or indicated, NeoSealer Flo had the most favorable filling for obturating anatomically complex apical regions.
